# Improving Ni^2+^ Tolerance of Arabidopsis by Overexpressing Bacterial *rcnA* Gene Encoding a Membrane-Bound Exporter of Ni^2+^

**DOI:** 10.3390/ijms26010227

**Published:** 2024-12-30

**Authors:** Xuxu Wang, Gengcheng Qiu, Jiading Yang

**Affiliations:** Co-Innovation Center for Sustainable Forestry in Southern China, College of Life Sciences, Nanjing Forestry University, Nanjing 210037, China; wangxuxu10298@163.com (X.W.); gcqiu@njfu.edu.cn (G.Q.)

**Keywords:** heavy metal, plant tolerance, nickel, rcnA, bacterial exporter, genetic transformation, phytoremediation

## Abstract

The prerequisite for breeding a plant to be used in phytoremediation is its high tolerance to grow normally in soil contaminated by certain heavy metals. As mechanisms of plant uptake and transport of nickel (Ni) are not fully understood, it is of significance to utilize exogenous genes for improving plant Ni tolerance. In this study, *rcnA* from *Escherichia coli* encoding an exporter of Ni and cobalt was overexpressed constitutively in *Arabidopsis thaliana*, and the performance of transgenic plants was assayed under Ni stress. The subcellular localization of rcnA in plant cells was found to be the plasma membrane. Constitutive overexpression of *rcnA* in Arabidopsis rendered better growth of either seedlings on agar medium containing 85, 100, and 120 μM NiCl_2_ or adult plants in a nutrient solution with 5 mM NiCl_2_ added. Compared to the wildtype, *rcnA*-OE transgenic plants under Ni stress accumulated lower levels of reactive oxygen species (i.e., superoxide and hydrogen peroxide) in leaves and exhibited less oxidative damage in shoots, as demonstrated by less electrolyte leakage and the lower malondialdehyde content. Notably, *rcnA*-OE transgenic plants retained a higher content of Ni in roots and had a lower content of Ni in shoots. Therefore, our findings indicated that the bacterial *rcnA* gene may be utilized to improve plant Ni tolerance through genetic transformation.

## 1. Introduction

The acceleration of industrialization and urbanization has resulted in an increasing area of soil contaminated by heavy metals (HMs), which may lead to adverse impacts on both human health and the environment [1]. Nickel (Ni) is one of the 23 metallic pollutants that make up 3% of the total composition of the Earth [2]. It has been reported that approximately 30,000 tons of nickel per year are emitted into the atmosphere from its natural sources and then accumulate in soil [3]. On the other side, the large growth in the nickel industry and consumption of nickel-containing products has inevitably intensified environmental Ni contamination by releasing nickel elements and varieties of its by-products. In the last decades, Ni contamination has been reported from sites all over the world, including Asia, Europe, and North America [4]. In China, a national survey showed that 4.8% of farmland soil was contaminated with Ni, making Ni the second highest soil pollutant, just following cadmium (Cd) [5].

Although Ni is essential for plant growth and is a constituent of several metalloenzymes, such as urease, Ni-Fe hydrogenase, and Ni-superoxide dismutase [6], only trace Ni is needed by plants, and nickel found in soils is usually sufficient for most plant species [7]. Excessive Ni entering plants from soil may disturb root elongation and nutrient uptake, inhibit photosynthesis and transpiration activities, render leaf chlorosis, and induce cell and tissue necrosis [8], thus resulting in reduced seed germination, plant growth, and biomass production. Especially, as a mobile element, crops grown in Ni-contaminated soil may accumulate high concentrations of Ni in edible parts [9], which may result in excessive Ni exposure in the human body via food consumption and pose a serious threat to human health [10]. There is an urgent need to reduce Ni concentrations in industrial and sewerage-contaminated surface soil, especially when it is utilized for crop cultivation.

Excessive Ni in soil and its toxicity could be alleviated by various remediation techniques through physicochemical and/or biological processes [11]. As a widely accepted low-cost and eco-friendly method of soil remediation, phytoremediation involves growing plants on Ni-contaminated soils and attaining a substantial reduction in Ni content and toxicity [12]. The ideal plants used for phytoremediation are those with the capacity to grow normally in Ni-contaminated soil and produce substantial biomass containing high Ni concentration. With several decades of investigation, hundreds of Ni hyperaccumulator plants have been identified to accumulate Ni in their living parts to levels being hundreds or thousands of times higher than most plants [13]. These Ni hyperaccumulator plants are mainly small herbaceous species from the families Asteraceae and Brassicaceaein and several tropical woody tree species in the genus *Phyllanthus* (Phyllanthaceae) distributed in Brazil, Cuba, New Caledonia, and Southeast Asia [14]. The limitation of the known Ni hyperaccumulator plants, such as low biomass production and/or strict terrestrial distribution, makes them unsuitable for being utilized in other geographical regions at a large scale for phytoremediation. It is of great significance to identify indigenous Ni hyperaccumulator plants or to genetically modify certain local species to improve their capacity for Ni extraction from soil.

The plant root systems absorb Ni in ionic form from the soil by both active and passive mechanisms [15]. For a certain species, the ratio of active and passive Ni transport may depend on the Ni concentration, pH of the growth media, presence of organic matter or other ions in soil or nutrient solution [12]. The absorbed Ni^2+^ is transported to xylem tissues in the transpiration stream and delivered to shoots and leaves, even to other plant parts like seeds, fruits, and buds via phloem tissues [16]. Besides the Ni concentration itself, Ni–ligand complexes (e.g., nicotianamine, histidine, organic acids, and certain proteins), which help in binding and transporting Ni, strongly influence Ni transport inside plants [12,17]. At the cellular level, the excessive Ni in cytoplasm induces the overproduction of reactive oxygen species (ROS) by inhibiting the activity of antioxidative systems, which may then cause a series of damages in cells, including membrane peroxidation, cytosol leakage, and chlorophyll bleaching [6,18].

Plants have evolved several potential mechanisms to protect against high concentrations of HMs including Ni. For different plant species, various strategies (e.g., reducing uptake, increasing vacuolar sequestration or excretion, maintaining high anoxidative activity, and enhancing the production of defense proteins) may be utilized to alleviate Ni toxicity, thus exhibiting certain tolerance to high Ni present in soil [19,20,21]. In a previous study comparing Ni contents of vacuoles from leaves of Ni hyperaccumulator *Thlaspi goesingense* and from the non-tolerant non-accumulator *Thlaspi arvense*, it was found that *T. goesingense* accumulated approximately 2-fold more Ni in the vacuole than the non-accumulator *T. arvense* under Ni exposure conditions [22]. In leaves of a hyperaccumulator *Hybanthus floribundus*, Ni was found to be predominantly localized in vacuoles of epidermal cells and outside of cell walls throughout the leaves [23]. As for two halophytes (i.e., *Sesuvium portulacastrum* and *Cakile maritima*) that were cultured hydroponically in various solutions of NiCl_2_, *S. portulacastrum* was found to have a higher tolerance to Ni, as demonstrated by better photosynthesis parameters, and consistently, *S. portulacastrum* accumulated a higher Ni concentration in shoots, with a greater percentage in cell walls but a smaller percentage in the soluble fraction, compared to *C. maritima*, indicating that a better ability to sequester Ni in the cell walls may be responsible for the higher Ni tolerance of *S. portulacastrum* [24]. It is conceptually accepted that intensifying the capacity of extracellular excretion or vacuolar sequestration to maintain low concentrations of toxic metals such as Ni in the cytoplasm may be beneficial for plant performance under excessive Ni conditions [8,25]. However, the chemical basis of Ni sequestration by cell walls in plants is poorly known, and no specific Ni efflux transporter has been identified in plants [6,8], which thus limits our attempt to improve plant Ni tolerance via modifying this aspect.

Similar in plants, nickel is an essential element for many microorganisms including *Escherichia coli*, in which Ni may be a cofactor for several enzymes including urease and [NiFe]-hydrogenase [26]. Because Ni at high concentrations can be toxic to microbes by displacing the cognate metals and making metalloprotein inactive [27], the Ni concentration in cells is tightly controlled and regulated. *E. coli* has a unique membrane-bound protein rcnA for exporting Ni and cobalt (Co), and mutation of *rcnA* increased the accumulation of nickel inside the cell and rendered the *E. coli* mutant more sensitive to Ni(II) and Co(II) [28]. In this study, we tested the potential of the *E.coli rcnA* gene as a molecular tool for increasing plant Ni tolerance by promoting Ni export in plant cells. We generated *rcnA*-overexpressing transgenic Arabidopsis plants and showed that these transgenic plants had a higher Ni tolerance under both agar medium and solid mixture growth conditions. This study thus may provide useful insight for breeding plants utilized for phytoremediation of Ni-contaminated soil through genetic engineering.

## 2. Results

### 2.1. Subcellular Localization of rcnA in Plant Cells

**Figure 1:**
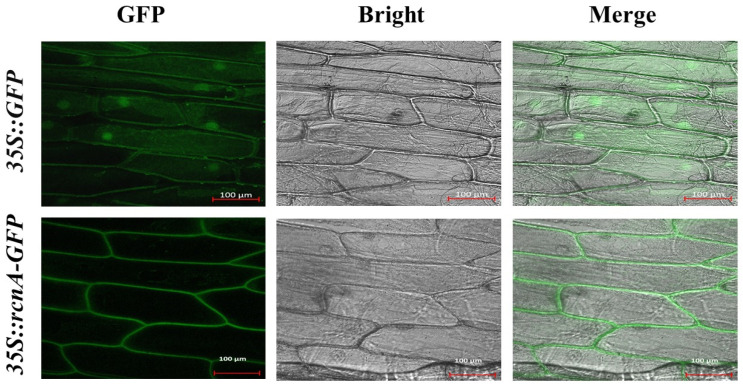
Subcellular localization of rcnA protein in plant cells. **Top** row showed the localization of free GFP protein in onion epidermal cells transformed with *35S::GFP* control vector. The **Bottom** row showed the localization of rcnA-GFP fusion protein in cells transformed with *35S::rcnA-GFP* construct. GFP, Bright, and Merge above figures represent GFP fluorescence images, bright-field images, and merged GFP and bright-field images, respectively. The scale bar represents 100 μm. Multiple cells were observed with similar results and the representative pictures were exhibited.

To determine the subcellular location of rcnA protein when it was expressed in plant cells, a translational fusion of rcnA and green fluorescent protein (GFP) was constructed under the control of the constitutive *CaMV-35S* promoter (i.e., *35S::rcnA-GFP*). Compared to onion epidermal cells expressing the *35S::GFP* control vector in which the green fluorescence was detected in multiple compartments, including the nuclei, plasma membrane, and cytoplasm (Figure 1, top row), the GFP fluorescence in onion epidermal cells transformed with *35S::rcnA-GFP* was detected only in the plasma membrane (Figure 1, bottom row). This result indicated that bacterial rcn A, when expressed in plant cells, was localized in the plasma membrane. This was consistent with the prediction using the online software Plant-mPLoc [29], which showed that the cellular localization of rcnA in plant cells was the cell membrane.

### 2.2. Phenotype of Transgenic Arabidopsis Plants Grown on Ni-Containing Medium

**Figure 2:**
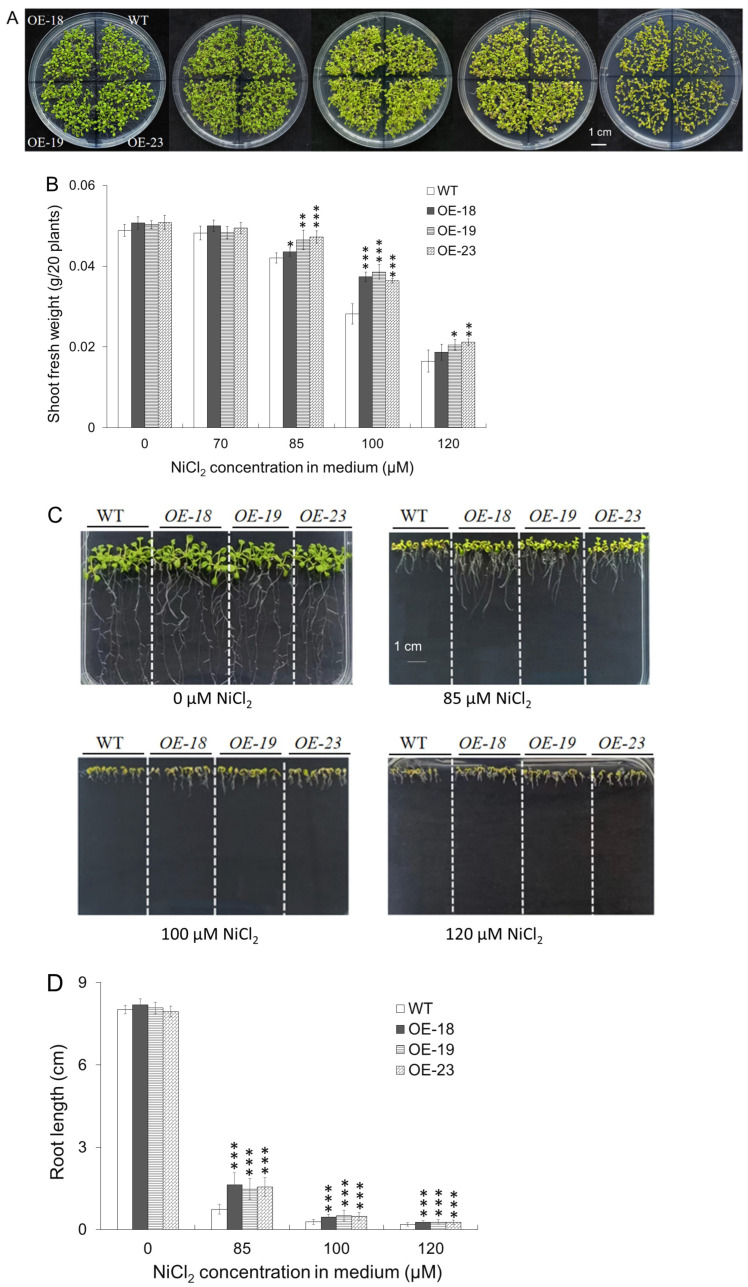
Phenotypes of *rcnA*-OE transgenic and wildtype Arabidopsis on half-strength MS medium containing various concentrations of NiCl_2_. Phenotype of plants and fresh weight of 20 seedlings grown horizontally for two weeks (**A**,**B**). Root phenotype and primary root length of plants grown vertically for two weeks (**C**,**D**). Asterisk *, **, and *** in (**B**,**D**) indicate a significant difference between a transgenic line and WT at *p* < 0.05, *p* < 0.01, and *p* < 0.001, respectively.

Through agrobacterium-mediated floral dip transformation, a total of 24 positive transgenic Arabidopsis T1 plants were identified by PCR to have insertion of *35S::rcnA* transgene (Supplementary Figure S1A). Most transgenic lines exhibited higher expression levels of *rcnA* than the wildtype (Supplementary Figure S1B). Three lines, i.e., OE-18, -19, and -23, that showed no phenotypic abnormality were selected for the physiological test. As shown in Figure 2A, along with increasing the Ni concentration from 0 to 120 μM in half-strength MS medium, the color of cotyledons and leaves changed from blue-green to yellow sequentially. Although there was no phenotypic difference between the transgenic lines and the wildtype on medium with 0 and 70 μM NiCl_2_, three transgenic showed apparent better growth than the wildtype on medium containing 85, 100, and 120 μM NiCl_2_, respectively. Both transgenic and wildtype plants died after three weeks on the media with 120 μM NiCl_2_. The shoot fresh weight on horizontal media plates showed a trend of declining with increasing of Ni concentration, and transgenic lines had a significantly higher fresh weight than the wildtype under 85, 100, and 120 μM NiCl_2_, except that OE-18 at 120 μM NiCl_2_ (Figure 2B). At 100 μM NiCl_2_, there exhibited the largest difference in shoot fresh weight between transgenic plants and the wildtype. When the wildtype and transgenic lines were grown on vertical media plates (Figure 2C), the root growth of either transgenic or wildtype Arabidopsis was remarkably inhibited by Ni treatment, while transgenic lines developed significantly longer primary roots than the wildtype in Ni-containing medium (Figure 2D). Especially, the largest difference of primary root length between transgenic plants and the wildtype was exhibited at 85 μM NiCl_2_. The better growth of both shoots and roots of transgenic plants on medium containing 85 μM and higher NiCl_2_ indicated that overexpression of *rcnA* in Arabidopsis improved Ni tolerance.

### 2.3. Contents of Anthocyanin and Malondialdehyde (MDA) in Shoots of Plants Grown on Horizontal Medium

**Figure 3:**
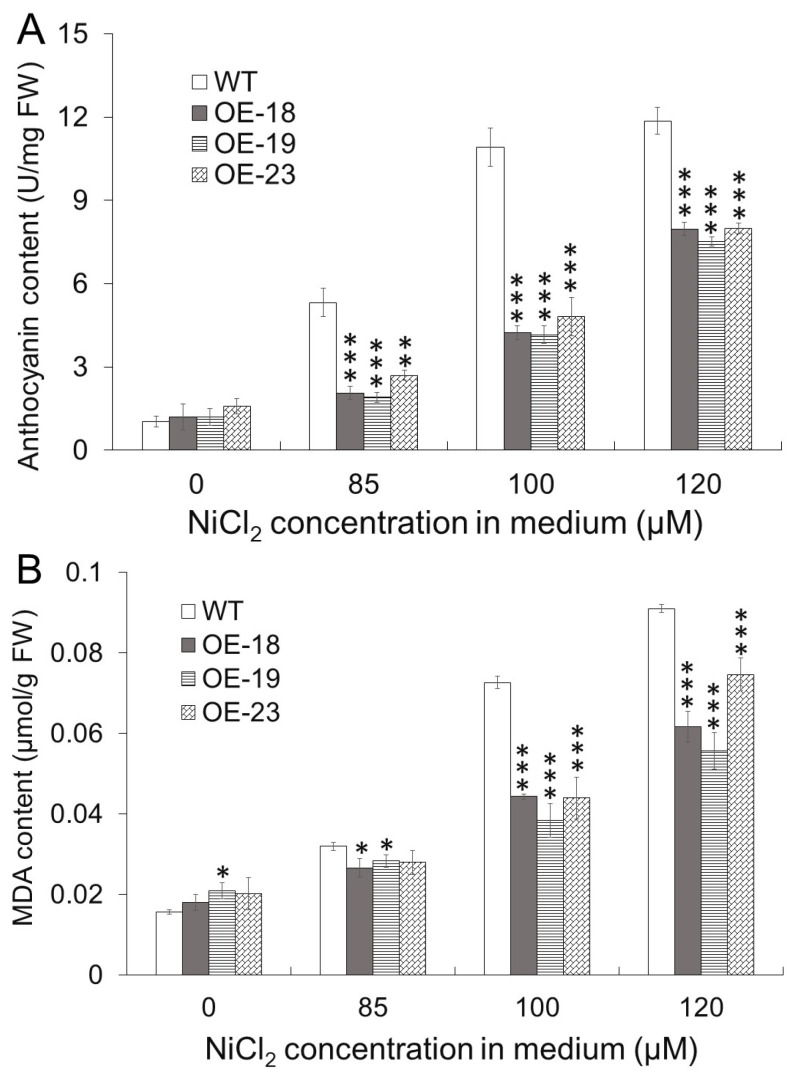
Contents of anthocyanin (**A**) and malondialdehyde (MDA) (**B**) in shoots of wildtype and three transgenic lines on medium supplemented with NiCl_2_. Arabidopsis seedlings were grown horizontally on medium for two weeks, and shoots were collected for measurement of anthocyanin and MDA. Asterisk *, **, and *** indicate significant difference between a transgenic line and WT at *p* < 0.05, *p* < 0.01, and *p* < 0.001, respectively.

The contents of anthocyanin and malondialdehyde were measured in shoots of Arabidopsis plants grown on horizontal medium containing 0, 85, 100, and 120 μM NiCl_2_, respectively. As shown in Figure 3A,B, both contents of anthocyanin and MDA in either the wildtype or *rcnA*-OE transgenic lines were increased along with increasing of Ni concentrations in the medium. For wildtype plants on medium with 85, 100, and 120 μM NiCl_2_, the anthocyanin contents were 5.1-, 10.5-, and 11.4-folds, respectively, as that on the media without Ni addition. But anthocyanin contents in *rcnA*-OE transgenic lines were significantly lower than that in the wildtype at a certain Ni concentration (Figure 3A). A similar trend was also found for MDA contents; i.e., transgenic lines accumulated less MDA than the wildtype when both were grown on medium containing NiCl_2_ (Figure 3B), indicating a lesser extent of membrane lipid peroxidation in transgenic lines under Ni treatment.

### 2.4. Comparison of Wildtype and Transgenic Arabidopsis Plants Grown Semi-Hydroponically

**Figure 4:**
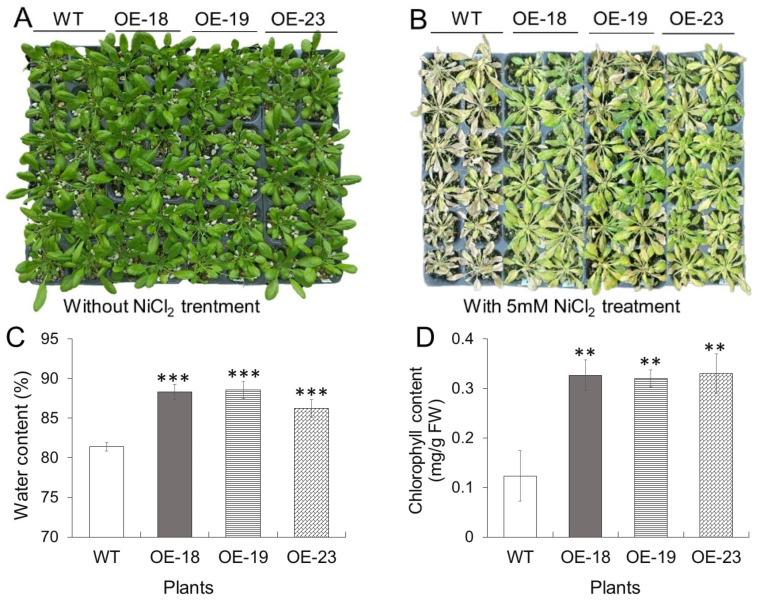
Wildtype (WT) and *rcnA*-OE transgenic Arabidopsis plants grown semi-hydroponically for two weeks. Phenotype of plants grown in control nutrient solution without NiCl_2_ addition (**A**) and with 5 mM NiCl_2_ (**B**). Natural water content (**C**) and chlorophyll content (**D**) in leaves of WT and transgenic plants irrigated with nutrient solution containing 5 mM NiCl_2_ for two weeks. Values in (**C**,**D**) were means ± SD. Asterisk **, and *** in (**C**,**D**) indicate significant difference between a transgenic line and WT at *p* < 0.01, and *p* < 0.001, respectively.

As shown in Figure 4A, there was no visible phenotypic difference between wildtype and *rcnA*-OE transgenic lines when grown in a nutrient solution without adding NiCl_2_. Both wildtype and transgenic seedlings became yellow and wilted after two weeks of treatment in a nutrient solution containing 5 mM NiCl_2_, and transgenic lines showed a less stressed phenotype of growth, compared to wildtype (Figure 4B). With 5 mM NiCl_2_ treatment, transgenic lines had significantly higher contents of water and chlorophyll than the wildtype (Figure 4C,D). On the contrary, the electrolyte leakage and malondialdehyde (MDA), both of which indicate the damage level of the cell membrane after stresses, were significantly higher in the wildtype than those in transgenic plants (Supplementary Figure S2A,B). These results indicated that *rcnA*-OE transgenic plants had an improved tolerance to exogenous Ni treatment.

### 2.5. Reactive Oxygen Species (ROS) Accumulation and Antioxidative Enzyme Activity

**Figure 5:**
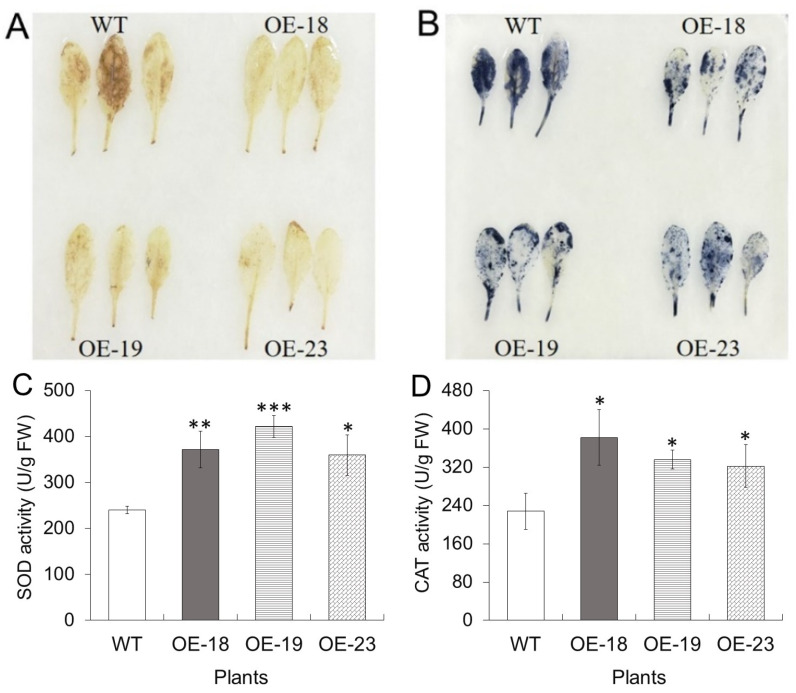
Accumulation of reactive oxygen species and activity of antioxidative enzymes in leaves of wildtype and *rcnA*-OE transgenic plants with 5 mM NiCl_2_ treatment for two weeks. Hydrogen peroxide accumulation exhibited by 3,3′-diaminobenzidine staining (**A**) and superoxide production exhibited by nitro blue tetrazolium staining (**B**). The experiments were repeated two times with similar results. Activities of superoxide dismutase (SOD) (**C**) and catalase (CAT) (**D**). Values in (**C**,**D**) were means ± SD. Asterisk *, **, and *** in (**C**,**D**) indicate significant difference between a transgenic line and WT at *p* < 0.05, *p* < 0.01, and *p* < 0.001, respectively.

To investigate the extent of oxidative stress induced by Ni treatment, the accumulation of two types of reactive oxygen species (ROS), i.e., superoxide and hydrogen peroxide (H_2_O_2_) in wildtype and *rcnA*-OE transgenic leaves, were estimated by histochemical staining methods [30]. As shown in Figure 5A, with 3,3′-diaminobenzidine (DAB) staining, wildtype leaves exhibited larger brown patches than transgenic leaves, indicating more H_2_O_2_ production in the wildtype under Ni treatment. Similarly, there was more intense blue staining on wildtype leaves than on transgenic leaves, indicating a higher production of superoxide in the wildtype.

### 2.6. Ni Content in Shoots and Roots of Arabidopsis Plants with Ni Treatments

**Figure 6:**
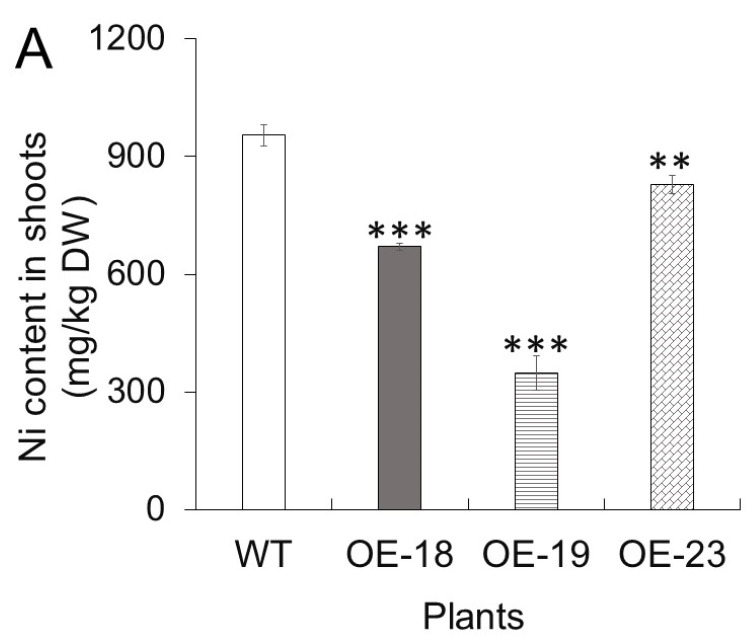

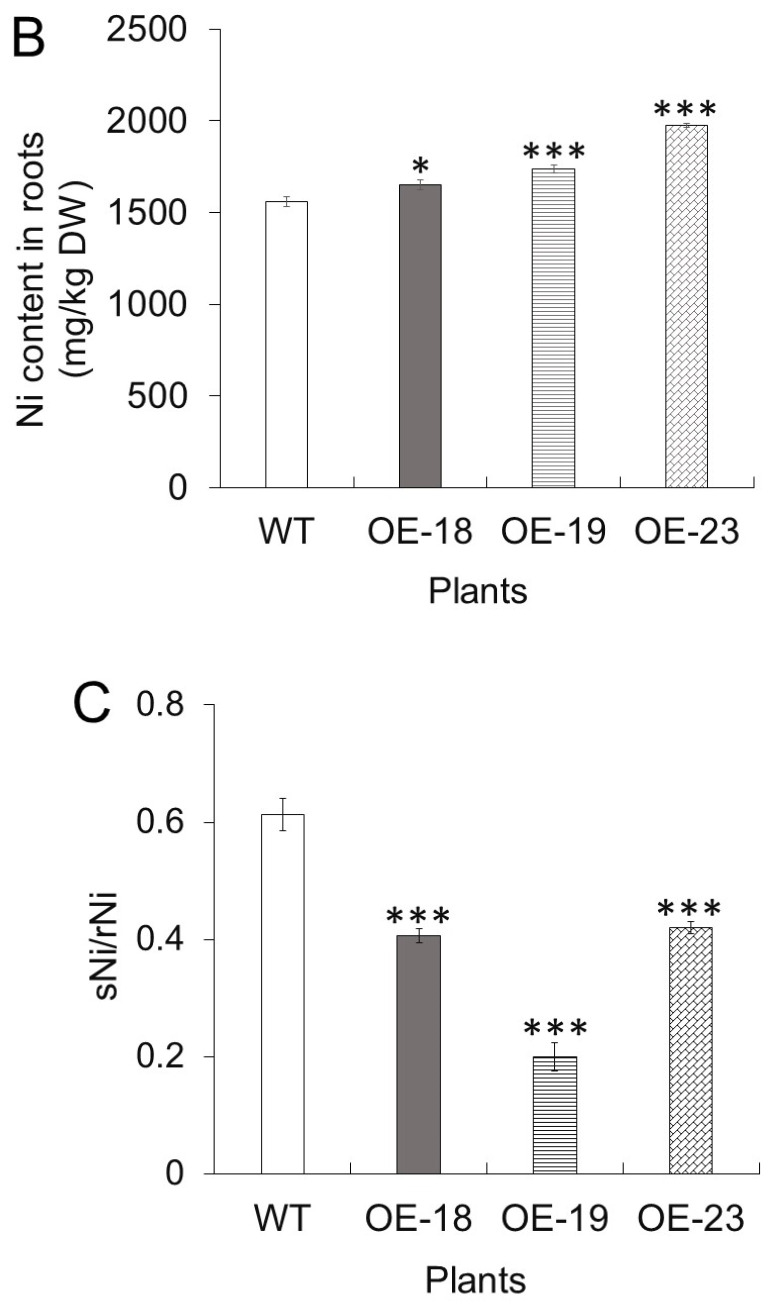
Ni contents in wildtype and three *rcnA*-OE transgenic Arabidopsis lines. Plants were grown in nutrient solution containing 5 mM NiCl_2_ for two weeks. The shoots and the roots were harvested, and the Ni contents were measured using ICP-MS. Ni content in shoots (sNi) (**A**), Ni content in roots (rNi) (**B**), and ratio of shoot Ni content and root Ni content (sNi/rNi) (**C**). The error bars represent SD. Asterisk *, **, and *** indicate significant difference between a transgenic line and WT at *p* < 0.05, *p* < 0.01, and *p* < 0.001, respectively.

To know if overexpression of *rcnA* could alter Ni accumulation or distribution in plants, the Ni contents were measured in the shoots and roots of Arabidopsis plants grown with 5 mM NiCl_2_. As shown in Figure 6A,B, the Ni content in the shoots of three transgenic lines was significantly lower, while the Ni content in transgenic roots was significantly higher than that in the wildtype. The smaller ratios of shoot Ni content and root Ni content (sNi/rNi) in three *rcnA*-OE transgenic lines meant that overexpressing *rcnA* in Arabidopsis resulted in more Ni retention in roots but attenuated Ni translocation to shoots (Figure 6C).

## 3. Discussion

The prerequisite for a plant to be used in phytoremediation is its capacity to grow normally in soil contaminated by certain HMs. Various breeding strategies could be utilized to improve plant tolerance to HMs, such as reducing uptake, increasing vacuolar sequestration or extracellular excretion, maintaining high anoxidative activity, etc. [21,22]. Early studies have demonstrated that ectopic expression of bacterial proteins involved in mercury metabolism could be used in improving plant resistance to organomercurials and mercuric ions [31,32]. The *merC* gene from *Acidithiobacillus ferrooxidans* encodes an inner membrane-spanning protein transporting mercuric ions into the cytoplasm [33,34]. Transgenic Arabidopsis and tobacco plants with constitutively expressing *merC* became hypersensitive to Hg^2+^ and accumulated approximately twice as much Hg^2+^ ion, relative to wildtype plants [35]. In our present study, as rcnA is a membrane-bound protein with the function of exporting Ni and cobalt (Co) in *E. coli* [28], we wonder if *rcnA* may be utilized to translocate Ni out of plant cells, thus, to decrease cytoplasmic Ni accumulation in plants and improve plant tolerance to soil excessive Ni.

Based on the observation of localization of rcnA-GFP fusion protein, we confirmed that rcnA protein in plant cells was primarily localized to the plasma membrane (Figure 1). As protein function is critically determined by its subcellular localization [36], rcnA being localized to the plasma membrane might support its putative role of exporting Ni from the cytoplasm. Three transgenic Arabidopsis lines with a relatively higher expression of *rcnA* (i.e., OE-18, -19, and -23) were selected to grow, together with the wildtype, on horizontal half-strength MS medium containing various concentrations of NiCl_2_ (Figure 2A), and transgenic lines showed better growth than the wildtype on medium with 85, 100, and 120 μM NiCl_2_, as demonstrated by the shoot fresh weight (Figure 2B). Furthermore, transgenic lines developed longer roots on vertical medium containing 85, 100, and 120 μM NiCl_2_ (Figure 2C,D). Under various abiotic stresses including HMs, anthocyanin and malondialdehyde (MDA) are induced in plant tissues, and their content may indicate the severity of stresses [37,38]. Compared to the wildtype, significantly lower levels of both anthocyanin and MDA in transgenic lines grown on medium with NiCl_2_ indicated alleviated cellular damage in shoots (Figure 3A,B). Under semi-hydroponic conditions, although wildtype and transgenic Arabidopsis adult plants showed no phenotypic difference when growing in the nutrient solution without Ni addition (Figure 3A), transgenic lines grew visibly better than the wildtype after two weeks of growing in the nutrient solution containing 5 mM NiCl_2_ (Figure 3B). Relative to the wildtype, transgenic plants had significantly higher water content and chlorophyll content (Figure 4C,D). These results collectively demonstrated that *rcnA*-OE transgenic Arabidopsis plants had an improved Ni tolerance than the wildtype under either agar medium or semi-hydroponic growth conditions.

Excessive accumulation of Ni in plants usually disturbs the balance of ROS producing and scavenging, leading to the accumulation of ROS, which may result in oxidative damage, including membrane lipid peroxidation and electrolyte leakage [6,39]. In the present study, accumulation of main ROS, i.e., superoxide and hydrogen peroxide (H_2_O_2_), was estimated by histochemical staining, and it was found that transgenic leaves exhibited less both brown staining of H_2_O_2_ and blue staining of superoxide (Figure 5A,B), i.e., lower accumulation of H_2_O_2_ and superoxide, respectively. Consistently, shoots of transgenic plants were found to have lower electrolyte leakage and lower content of malondialdehyde (MDA), which is one of the final products of lipid peroxidation [40] (Supplementary Figure S2). Considering that the steady-state level of superoxide radicals and H_2_O_2_ is mainly determined by the balance between activities of superoxide dismutase SOD and H_2_O_2_-degrading enzymes (such as CAT) [41], the activities of SOD and CAT were measured and were found to be significantly higher in three transgenic plants than in the wildtype under Ni treatment (Figure 5C,D). As rcnA is a Ni exporter and should not have a direct regulatory role in protein activity or gene expression, the higher activities of SOD and CAT in transgenic plants with Ni treatment might be attributable to less inhibition of antioxidative enzymes by the lower Ni content in transgenic shoots (Figure 6A).

The root is the first plant organ to contact with and absorb HMs from soil or medium, and most plants tend to accumulate toxic HMs more in the roots than in the shoots [42]. In this study, for both wildtype and transgenic plants, the Ni content in roots was much higher than that in shoots (Figure 6A,B). Notably, *rcnA*-OE transgenic plants contained higher Ni content in roots but lower Ni content in shoots than the wildtype, though both plants were grown with the same Ni treatment. The smaller ratios of shoot Ni content and root Ni content (sNi/rNi) in three transgenic plants indicated a lower percentage of Ni being translocated from roots to shoots (Figure 6C), which may thus induce less ROS production and oxidative stress (Figure 5, Supplementary Figure S2) and render better shoot growth in transgenic plants (Figure 4). Excessive HMs usually result in decreased elongation of the primary root and impaired secondary growth [43]. In the present study, when both were grown on the same agar medium containing 85, 100, and 120 μM NiCl_2_, transgenic lines exhibited longer roots, indicating less inhibitory effect imposed by NiCl_2_ compared to wildtype (Figure 2C,D). It thus indicated that overexpression of *rcnA* could improve tolerance of Arabidopsis roots to exogenous Ni and render a relatively better growth.

Subcellular reallocation of HMs, which are imported into cells, is an internal mechanism for plants to avoid toxicity and may determine plant tolerance to HMs [44]. In rapeseed, two genotypes with opposite resistance to Cd were found to contain similar Cd concentrations in both whole plants and within individual tissues, but more Cd was accumulated in the shoot vacuoles and root cell walls of the resistant genotype than in the respective subcellular compartments of the sensitive genotype [45]. Similar results were reported previously, as in *S. portulacastrum*, which showed a higher tolerance to Ni and a greater percentage of Ni in the cell walls of shoots compared to *C. maritima* with a lower Ni tolerance [24], and in two *Thlaspi* plants that the hyperaccumulator species accumulated approximately 2-fold more Ni in the vacuole than the non-accumulator [22]. Interestingly, when a bacterium mercury uptake pump *merC* was overexpressed in Arabidopsis, the transgenic plants grew significantly less well on an HgCl_2_-containing media, indicating the reduced tolerance to HgCl_2_ [35]. Although merC and rcnA exhibited the same subcellular localization as the plasma membrane in plant cells, their opposite function (i.e., merC as Hg importer versus rcnA as Ni exporter) rendered the opposite growth phenotype of transgenic plants (i.e., reduced Hg tolerance of *merC*-overexpressing plants versus improved Ni tolerance of *rcnA*-overexpressing plants) (Figure 2 and Figure 4) [35]. Although measurement of Ni content in various cellular fractions of transgenic Arabidopsis roots in the present study was not performed due to instrumental limitation, based on the better growth of *rcnA*-OE transgenic plants under Ni stress and the rcnA function of exporting Ni across the plasma membrane, it may be reasonable to predict a higher Ni accumulation in extracellular space and thus lower Ni in the cytoplasm in *rcnA*-OE transgenic roots than in the wildtype. This may contribute to a decreased transportation of Ni to shoots in *rcnA*-OE transgenic plants (Figure 6A,B). Thinking from the view of breeding plant cultivars for soil phytoremediation, the lower accumulation of Ni in shoots may not be desired because it may reduce the efficiency of removing soil Ni by harvesting aboveground tissues [46]. However, considering that sequestrating HMs in the vacuole is another mechanism underlying plant tolerance to HMs, it is of interest to modify the subcellular localization of rcnA and investigate if the transgenic plants with rcnA being localized in the tonoplast could grow better and accumulate higher Ni levels in shoots under excessive Ni stress. The result might be instructive for breeding plant cultivars used for the phytoremediation of soil Ni.

In summary, this study confirmed the potential of the bacterial *rcnA* gene encoding Ni exporter in improving plant Ni tolerance. It was found that the subcellular localization of rcnA in plant cells was the plasma membrane. Constitutive overexpression of *rcnA* in Arabidopsis rendered better growth of either seedlings on certain NiCl_2_-containing medium or adult plants in nutrient solution with NiCl_2_ addition. *RcnA*-OE transgenic plants under Ni stress exhibited lower ROS accumulation and less oxidative damage in shoots. Transgenic plants retained more Ni in roots and translocated less Ni to shoots. Therefore, *rcnA* is functional when expressed in plant cells and may have the potential to be utilized to improve plant Ni tolerance through genetic transformation.

## 4. Materials and Methods

### 4.1. Plant Materials and Growth Conditions

*Arabidopsis thaliana* ecotype Colombia (Col-0) was used as wildtype for genetic transformation and used as control for phenotypic/physiological comparison. Arabidopsis seeds were surface-sterilized with 75% (*v*/*v*) ethanol. After incubation at 4 °C for 2 d, seeds were germinated and grown on half-strength Murashige and Skoog (MS) medium containing 0.8% agar and 1% sucrose for 7 d (16 h light and 8 h dark) with white light illumination (100 μmol/s/m^2^) at 22 °C and 55–60% relative humidity. For Ni treatment, the half-strength MS medium was supplemented with various concentrations of NiCl_2_. For semi-hydroponic growth, Arabidopsis plants were first germinated on half-strength MS agar plates for 7 days, and then the seedlings were carefully transplanted into the mixture of vermiculite: perlite: glass beads (with diameter of 0.3 cm) with a volume ratio of 1:1:1 and irrigated with half-strength Hoagland solution containing 5 mM NiCl_2_ from the bottom of tray.

### 4.2. Cloning rcnA Gene and Plasmid Construction for Plant Stable Transformation

The genomic DNA of *E. coli* DH5α was extracted by using TIANamp Bacteria DNA kit (Tiangen Biotech, Beijing, China) following the manufacturer’s standard protocols and used as template for polymerase chain reaction (PCR). The specific primers were designed according to the coding sequence of *rcnA* curated by EcoCyc *E. coli* Database [47].

For overexpression of *rcnA* in Arabidopsis, PCR was performed using primer pairs rA-F1 and rA-R1 in which the additional restriction endonuclease BglⅡ and PmlⅠ sites were added at 5′ ends, respectively (as listed in Supplementary Table S1). The amplified fragment was cloned into pCAMBIA1305m vector (without NcoⅠ site) through BglⅡ and PmlI to replace GUSPlus region, thus resulting in *35S::rcnA* construct, which was then confirmed by DNA sequencing. The *35S::rcnA* construct was transferred into *Agrobacterium tumefaciens* strain EHA105 by the freeze–thaw method [48], which was then used to transform Arabidopsis wildtype, following the floral dip method [49]. Transgenic T1 seeds were germinated on half-strength MS agar plates containing hygromycin (12 mg/L), and the resistant seedlings were transferred into soil for seed production. The genomic DNA was extracted from rosette leaves of multiple resistant plants for PCR identification of positive *35S::rcnA* transgenics, using primers 35S-inter-F and rA-R1. The total RNAs were extracted from young rosette leaves of various positive transgenic plants to measure the expression of *rcnA* via quantitative real-time PCR (qPCR). qPCR was performed following previous study [50], and Arabidopsis *UBQ10* (At4g05320) was used as internal control gene for calculating relative expression level of *rcnA* in transgenic Arabidopsis plants. Phenotypic analysis was performed on selected homozygous T3 transgenic *rcnA*-OE lines with high expression of *rcnA* and without obvious phenotypic abnormality.

### 4.3. Subcellular Localization of rcnA in Plant

For checking subcellular localization of rcnA in plant cells, the coding sequence of *rcnA* without stop codon TAA was PCR-amplified with specific primers rA-F2 and rA-R2 and cloned into pCAMBIA1302 vector via NcoⅠ and SpeI sites just upstream GFP report gene using homologous recombination. The resulting *35S::rcnA-GFP* construct was confirmed with correct opening frame for *rcnA-GFP* fusion protein by sequencing. The *35S::rcnA-GFP* construct or the *35S::GFP* control vector was transiently expressed in onion (*Allium cepa*) epidermal cells using an Agrobacterium-mediated method as report previously [51]. Subcellular localization of the GFP signal was observed using a confocal laser scanning microscope (LSM710, Carl Zeiss AG, Oberkochen, Germany). The subcellular localization of *rcnA* in plant cells was also predicted by online software Plant-mPLoc (http://www.csbio.sjtu.edu.cn/bioinf/plant-multi/ (accessed on 24 December 2024)) [29].

### 4.4. Determination of Growth on Medium Plates

Both wildtype and *rcnA*-OE transgenic Arabidopsis seeds were germinated and allowed to grow either horizontally or vertically on half-strength MS medium with added NiCl_2_ at 0, 70, 85, 100, and 120 μmol/L, respectively, for two weeks. Growth phenotype was observed, and parameters (i.e., shoot fresh weight and root length) were measured. The content of anthocyanin in shoots was measured by following previous methods with some modifications [52,53]. Briefly, about 0.1 g (fresh weight) of shoot samples was ground in liquid nitrogen and shaken gently in 0.6 mL 1% methanolic HCl solution (i.e., methanol:H_2_O:3M HCl at volume ratio of 75:24:1) in dark for 24 h at 4 ℃. The relative content of anthocyanin was calculated according to the absorptances of methanolic extracts at 530 nm and 630 nm as (A_530_ − 0.24 × A_630_). One unit of anthocyanin content was expressed as an absorptance change of 0.1. MDA measurement was performed as described previously, with minor modifications [54]. About 0.3 g (fresh weight) of shoot samples was ground in liquid nitrogen and extracted in 3 mL of 5% (*w*/*v*) trichloroacetic acid. The 2 mL supernatant after centrifugation at 10,000× *g* for 10 min at 4 ℃ was mixed with 2 mL 0.067% (*w*/*v*) thiobarbituric acid. The mixture was heated to 100 °C for 20 min, cooled quickly in an ice water bath, and centrifuged at 10,000× *g* for 10 min. The absorbances of supernatant at 532, 600, and 450 nm were measured, and MDA concentration (μmol/L) was calculated as 6.45 × (A_532_ − A_600_) − 0.56 × A_450_. The MDA contents in shoots were calculated based on sample fresh weight as μmol/g FW.

### 4.5. Growth and Physiological Parameters of Plants Under Semi-Hydroponic Condition

Both wildtype and selected *rcnA*-OE transgenic Arabidopsis lines were grown semi-hydroponically in half-strength Hoagland solution for three weeks, and then two parallel sets of plants were irrigated with half-strength Hoagland solution without and with containing 5 mM NiCl_2_, respectively, for two weeks. The phenotypes of wildtype and transgenic Arabidopsis were observed. For a certain line, all rosette leaves of four plants were put together as one shoot sample, and roots of the same plants were washed carefully and gently in ddH_2_O for three times to remove any attached particles or ions and were combined as one root sample after surface water on roots was absorbed with filter paper. There were three replicates for both leaf and root samples. The experiments were repeated two times.

### 4.6. Determination of Physiological Parameters of Rosette Leaves

The fresh and dry weight of leaf samples were used to calculate the natural water content (NWC) as (FW–DW)/FW. For chlorophyll content measurement, about 0.2 g leaf samples was ground into fine powder in liquid nitrogen and extracted in 3 mL of 80% acetone. The absorbances at 663 nm and 645 nm were determined to calculate chlorophyll contents according to our previous report [55]. Besides malondialdehyde (MDA) content, the plasma membrane damage in mature leaves was also estimated by relative electrolyte leakage [56]. For enzyme activity measurement, about 0.3 g leaf samples was homogenized in 3.0 mL of cold extraction buffer [50 mM phosphate buffer (pH 7.0) containing 1% (*m*/*v*) polyvinylpyrrolidone] with a mortar and pestle in an ice bath and centrifuged at 15,000× *g* for 15 min at 4 °C. The supernatant was used as raw enzyme solution. The activity of superoxide dismutase (SOD) in leaf samples was determined by measuring its ability to inhibit the photoreduction of nitro blue tetrazolium (NBT) [57]. One unit of SOD activity was defined as the amount of enzyme that would inhibit 50% of NBT photoreduction. The activity of catalase (CAT) was determined by monitoring absorbance at 240 nm, which indicates the changes in H_2_O_2_ concentration in enzymatic reaction [57]. The CAT reaction solution (3.0 mL) contained 50 mM phosphate buffer (pH 7.0) and 20 mM H_2_O_2_. The reaction was initiated by adding 0.1 mL of enzyme extract. One unit of CAT activity was calculated as an absorbance decrease of 0.01 per min.

### 4.7. Qualitative Determination of Superoxide and H_2_O_2_ Contents in Leaves

The accumulation of superoxide and H_2_O_2_ in mature rosette leaves was visually exhibited through nitro blue tetrazolium (NBT) staining and 3,3′-diaminobenzidine (DAB) staining, respectively [30]. The appearance of blue spots indicated the presence of superoxide, while brown spots indicated the production of H_2_O_2_. At least 3 leaves of a certain line were stained, and the experiments were repeated two times.

### 4.8. Determination of Nickel Content in Leaf and Root Samples

About 0.2–0.5 g (accurate to 0.0001 g) of dried leaf or root samples of each line was sent for nickel content measurement by Webiolotech Testing Company (Nanjing, China). The samples were digested completely in 5 mL of HNO_3_ in sealed Polytetrafluoroethylene digestion vessels, which were kept in a laboratory oven at 80 °C for 2 h, 120 °C for 2 h, then 160 °C for 4 h, and cooled down to room temperature. The digestion solutions were evaporated to dry by heating and then adjusted to 25 mL with 1.0% (*v*/*v*) HNO_3_. The concentration of Ni was analyzed with NexION 5000 Multi-Quadrupole Inductively Coupled Plasma-Mass Spectrometry ICP-MS (PerkinElmer, Shelton, CT, USA). The content of Ni in leaves or roots was calculated and expressed as mg/kg dry weight (DW).

### 4.9. Statistical Analysis

For those numerical parameters, the averages were presented with standard deviations (SD) of three biological replicates. To compare a transgenic line and control (wildtype), F-test in Microsoft Excel was first performed to evaluate whether they have equal variance, and then the two-tailed Student’s *t*-test embedded in Microsoft Excel was used to analyze whether their averages have a significant difference at *p* < 0.05, *p* < 0.01, and *p* < 0.001 levels, respectively.

## Data Availability

The original contributions presented in this study are included in this article/Supplementary Materials. Further inquiries can be directed to the corresponding author.

## Supplementary Materials

The following supporting information can be downloaded at https://www.mdpi.com/article/10.3390/ijms26010227/s1.
