# Supplementary material for: Improving Ni2+ Tolerance of Arabidopsis by Overexpressing Bacterial rcnA Gene Encoding a Membrane-Bound Exporter of Ni2+
Source: Int J Mol Sci. 2024 Dec 30;26(1):227. doi: 10.3390/ijms26010227 (PMC11719643; doi:10.3390/ijms26010227)
Supplement: Supplementary file 1 [file ijms-26-00227-s001.zip › ijms-3359874-supplementary.pdf]

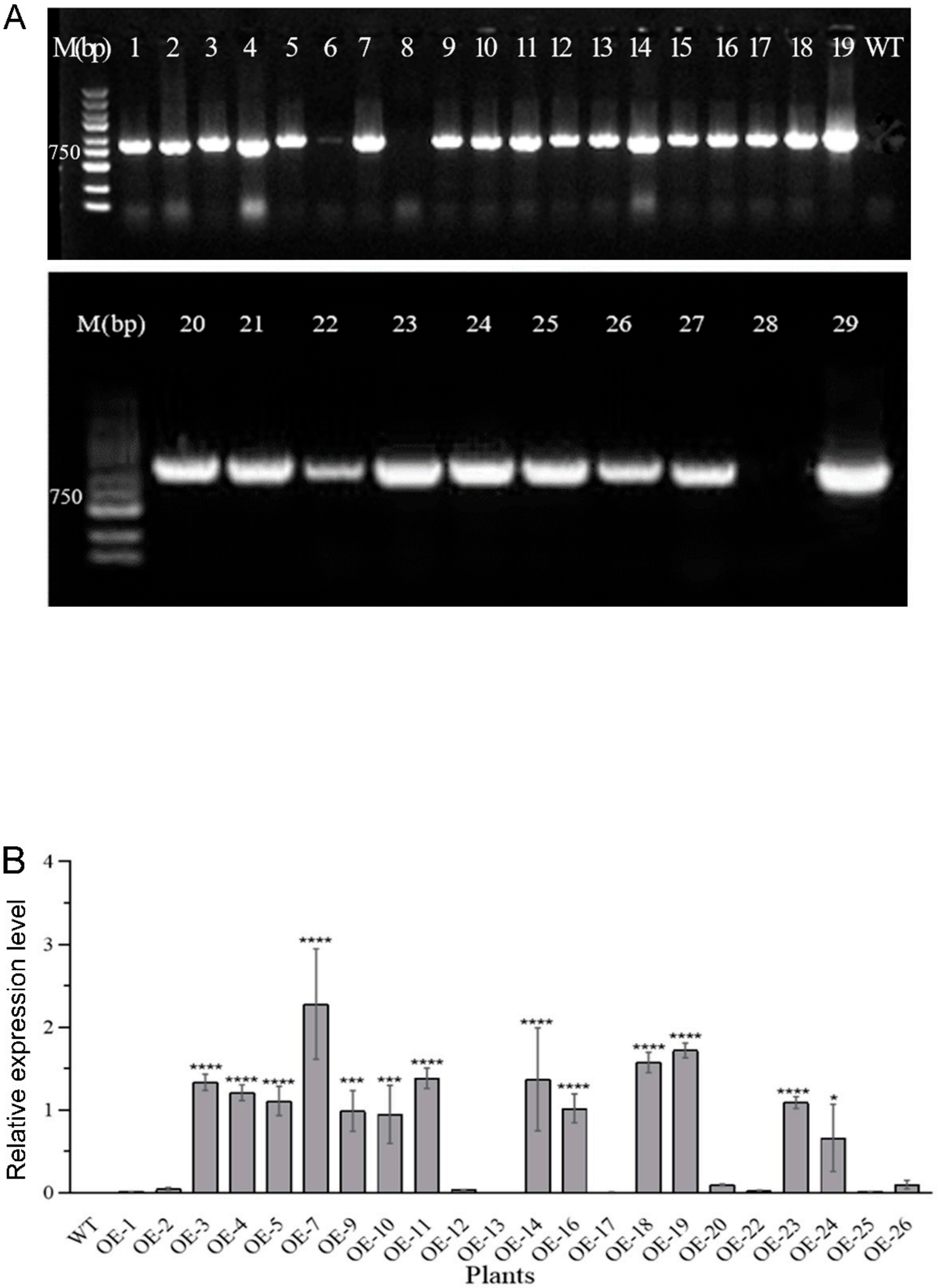

Figure S1 Positive transgenic lines identified by PCR (A). Relative expression levels of *rcnA* in various *35S::rcnA* transgenic lines (B).

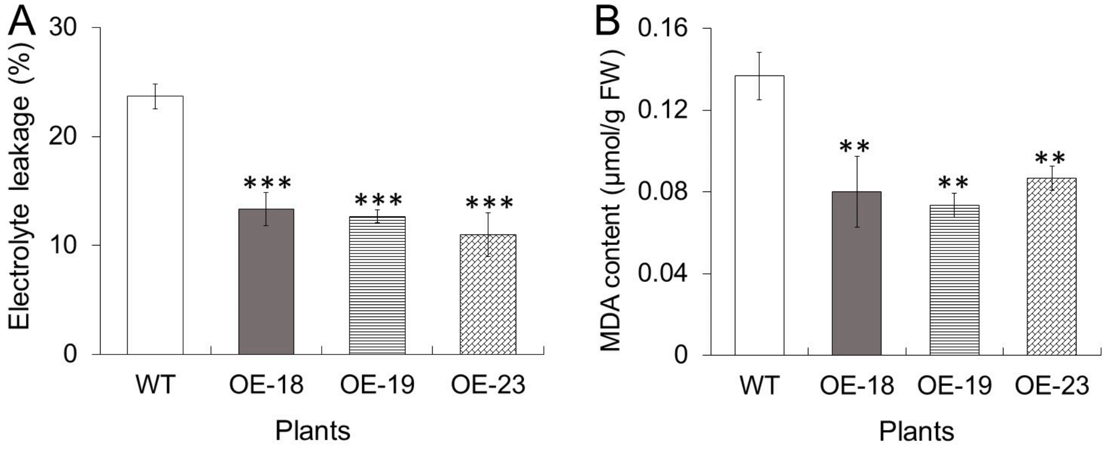

Figure S2 The electrolyte leakage (A) and malondialdehyde (MDA) content (B) in shoots of wildtype and *rcnA*-OE transgenic plants grown for two weeks in nutrient solution containing 5 mM $NiCl_2$. Values were means ± SD. Asterisk *, **, and *** indicate a significant difference between a transgenic line and WT at $p < 0.05$, $p < 0.01$, and $p < 0.001$, respectively, using the Student's t-test in Microsoft Excel.

Table S1 Primer sequences used in this study

**Name**	**sequence (5'→3')**	**Application**	**Note**
rA-F1	TCAGATCTATGACCGAATTTACAACTCTTCTT	amplifying coding sequence of *rcnA* for constructing *35S::rcnA*	BglII site as underlined
rA-R1	TTCACGTGTTATCGCATTATGCCCATG	amplifying coding sequence of *rcnA* for constructing *35S::rcnA*	PmlI site as underlined
rA-F2	acgggggactcttgaccatggGAATGACCGAATTTACAACTCTTCTTCA	amplifying coding sequence of *rcnA* for constructing *35S::rcnA-GFP*	nucleotides in lower case being homologous recombination arm
rA-R2	aagttcttctcctttactagtTCGCATTATGCCCATGAAGC	amplifying coding sequence of *rcnA* for constructing *35S::rcnA-GFP*	nucleotides in lower case being homologous recombination arm
qrA-F	GCTGGAGCGGATTTAACACT	qPCR measuring expression of *rcnA* in transgenic *35S::rcnA* Arabidopsis lines	
qrA-R	CCATGTACGCCCATATACACA	qPCR measuring expression of *rcnA* in transgenic *35S::rcnA* Arabidopsis lines	
qUBQ10-F	CACACTCCACTTGGTCTTGCGT	*AtUBQ10* (At4g05320) as qPCR internal control	
qUBQ10-R	TGGTCTTTCCGGTGAGAGTCTTCA	*AtUBQ10* (At4g05320) as qPCR internal control	
